# Endoscopic Submucosal Dissection for Resolution of a Symptomatic Pancreatic Rest in a Pediatric Patient: A Case Report and Literature Review

**DOI:** 10.1155/2021/8853120

**Published:** 2021-09-13

**Authors:** Sandra Mabel Camacho-Gomez, Chris Moreau, James Noel, Robert Adam Noel, Sandeep Patel

**Affiliations:** ^1^Department of Pediatrics, Division of Pediatric Gastroenterology, University of Mississippi Medical Center, Jackson, MS, USA; ^2^Department of Medicine, Division of Gastroenterology, University of Texas Health Science Center, San Antonio, TX, USA; ^3^Department of Pediatrics, Division of Pediatric Gastroenterology, Baylor College of Medicine at the Children's Hospital of San Antonio, San Antonio, TX, USA

## Abstract

The pancreatic rest, aberrant, or heterotopic pancreas is a normal function pancreas found in the submucosal layer of the greater curvature of the gastric antrum and occasionally in the duodenum. Most of the patients are asymptomatic and the finding is usually incidental. We describe the case of a child with abdominal pain and history of recurrent ulcers that necessitated esophagogastroduodenoscopy and further evaluation with endoscopic ultrasound that confirmed a submucosal lesion consistent with a pancreatic rest. Endoscopic submucosal dissection was performed without complication, and complete symptom resolution was achieved after dissection of the pancreatic rest.

## 1. Introduction

A pancreatic rest (PR) is heterotopic pancreatic tissue commonly found in the submucosal layer of the greater curvature of the gastric antrum and occasionally in the duodenum [1]. Most of the patients are asymptomatic and the finding is usually incidental [2]. The gastrointestinal symptoms most commonly associated with PR include abdominal pain, nausea, and vomiting [2]. We describe the case of a child with a symptomatic PR who experienced resolution of abdominal pain after an endoscopic submucosal dissection (ESD).

## 2. Case Presentation

**Figure 1:**
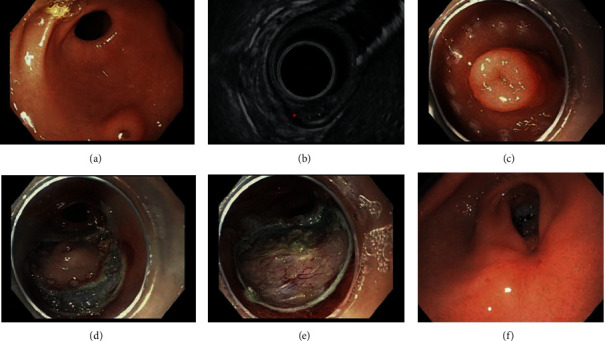
ESD of pancreatic rest and EUS. (a) Visualization of umbilicated lesion and gastric outlet. (b) EUS showing the contained submucosal mass (arrow) below scope. (c) Circumferential marking of region for ESD. (d) Circumferential dissection of lifted lesion. (e) Fully resected lesion. (f) EGD of area at 11 weeks after ESD.

A 7-year-old Hispanic female with a history of gastroesophageal reflux disease, recurrent ulcer disease, and vomiting presented with a chief complaint of constipation and second opinion of chronic pressure-like periumbilical abdominal pain. Before her visit, her pain was managed with a bland food diet and her ulcer disease was treated with pantoprazole, without improvement on her pain. Family history includes adenomatous polyps and gastric cancer. The parents reported no changes in appetite, vomiting, diarrhea, or weight loss. Physical exam was normal and polyethylene glycol 3350 was prescribed for constipation. She has a normal serum amylase and lipase. Esophagogastroduodenoscopy (EGD) was performed due to persistent symptoms and a 2 cm umbilicated lesion was identified along the posterior wall of the antrum of the stomach (Figure 1(a)). Biopsies were nondiagnostic. At 2-month follow-up, her abdominal pain remained unresolved. Due to familial and medical history, endoscopic ultrasound (EUS) examination of the lesion was suggested.

EUS revealed a mixed echogenic structure enclosing an anechoic structure consistent with PR (Figure 1(b)), no signs of inflammation. The structures were completely contained within the submucosal space, and an ESD was recommended. Our informed consent addressed the potential benefit of removing the PR for improving her response to conventional treatment for her chronic ulcer disease. We discussed that we had no guarantee that this would result in cure. We discussed the risks of ESD of the PR to include perforation, infection, and emergent surgery. The patient underwent ESD under general anesthesia. The lesion was marked circumferentially with a dual knife using forced coagulation settings (Figure 1(c)). A submucosal lift was created using 20 mL of injectable composition premixed with methylene blue. Circumferential mucosal resection was initiated at the markings using an electrosurgical knife with a dome-shaped cutting section and endocut electrocautery setting (Figure 1(d)). Submucosal tunneling was performed to complete the resection (Figure 1(e)), and minimal bleeding was controlled using soft coagulation setting. After ESD, the base of the lesion and ulcer appeared clean with no further bleeding.

**Figure 2:**
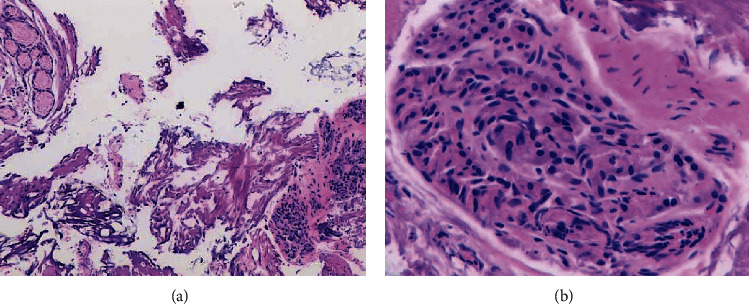
Hematoxylin and eosin stain showing PR: (a) PR (right) seen adjacent to gastric antral mucosa (left). (b) Pancreatic exocrine glands consistent with PR.

Excised tissue was captured with a net retriever tool and sent to pathology, which revealed focal pancreatic heterotopia with acini, ducts, and islet cells in the subepithelium covered with distorted gastric mucosa, and thermal artifactual; there was no sign of dysplasia or inflammation (Figure 2). The patient was admitted for 24-hour observation and discharged home with no complications. At 6-week follow-up, symptoms had resolved and pantoprazole was discontinued. Postresection EGD at 11 weeks showed almost complete healing of the mucosa (Figure 1(f)) with normal gastric biopsies. The patient remains free of abdominal pain and no recurrence of the persistent ulcers after one year postresection.

## 3. Discussion

The PR, known as aberrant pancreas, functions as a normal pancreas [3]. A PR does not have a true anatomical or vascular connection to the pancreas [1, 3]. They may be found incidentally on EGD and radiographic examinations [2]. A round mass with central umbilicated and smooth surface appears endoscopically within 3 to 4 cm of the pylorus, ranging from 0.5 millimeters to 3 centimeters [1]. Cold forceps biopsies are usually not diagnostic due to the submucosal origin of the tumor [4]. On radiographic study, when a central duct is present, it may be filled with barium [1].

Most of the patients are asymptomatic [3]. Less common manifestations and complications than previously described, included pancreatitis, upper gastrointestinal bleeding, and gastric outlet obstruction [4], as well as biliary obstruction and intestinal obstruction [5].

The increasing availability of EUS has facilitated a more thorough examination of the lesion before intervention. EUS aids in determining the layer of the stomach wall from which the PR arises and helps characterize the lesion [3]. Some of the characteristics assessed include size, echogenicity, smoothness of the border, internal features, vascularity, relationship with other organs, and the presence of adenopathy [3]. The aberrant pancreas appears as a heterogeneous lesion, which includes hypoechoic masses with scattered hyperechoic areas, indistinct margins within the wall, and sometimes a tubular structure within the lesion [3]. The limitation of the EUS is the availability of the expertise in the pediatric population [6].

Most of the patients are asymptomatic and do not necessarily require resection [3]. However, the differential diagnosis for gastric subepithelial lesions includes both benign and malignant potential tumors [7]. A PR can have adverse clinical implications and diagnosis should be made with caution in a child. The association of PR with malignancy degeneration has been described in the adult population [8].

Historically patients with symptomatic PR underwent surgery of the rest by wedge excision and pyloroplasty or by submucosal enucleation through a gastrostomy [2]. Novel endoscopic approaches like endoscopic mucosal resection (EMR) have been described, and compared with conventional surgery, endoscopic treatment has the advantage of being less invasive and a lower cost [9]. EMR is considered if the lesion arises from the mucosa and submucosa, located within the innermost 3 layers of the wall on the evaluation by EUS [4]. EMR is not recommended if the lesion arises from the muscularis propria, the 4th inner layer of the wall, due to the risk of perforation and incomplete resection [4]. Cap assisted EMR provides better visualization of the operative field by facilitating suction and snaring of the target lesion within a plastic cylinder surrounding the tip of the endoscope, but is limited to lesions with a diameter less than 15 mm [4]. ESD can achieve *en bloc* resection of lesions regardless of tumor size [10]. The procedure is usually longer than the EMR and increases risk of bleeding and perforation [10]. Short-term proton-pump inhibitors (PPI) could be considered to help healing of the resulting ulcer [11].

In conclusion, we demonstrated a symptomatic pediatric patient with a PR successfully managed with ESD. Historically, clinical symptoms warrant surgical management of the PR. Abdominal pain unresponsive to PPI and history of recurrent ulcers in this patient necessitated EGD and further evaluation with EUS, which diagnosed with a submucosal lesion consistent with a PR. ESD was performed without complication, and complete symptom resolution was achieved after dissection of the PR.

## 4. Take-Away Lessons

A pancreatic rest is heterotopic pancreatic tissue commonly found in the submucosal layer of the stomach. Most of the patients are asymptomatic and the gastrointestinal symptom most commonly associated with pancreatic rest is abdominal pain. Patients usually do not necessarily require resection. However, the differential diagnosis for gastric subepithelial lesions includes tumor. Endoscopic mucosal resection is described as an endoscopic treatment with the advantage of being less invasive and at a lower cost, in the other hand; the endoscopic submucosal dissection can be performed regardless of the size of the tumor.

## Data Availability

No data were used to support this study.

## Additional Points

To our knowledge, there is no previous case report. Historically, patients with symptomatic pancreatic rest underwent surgery of the rest by wedge excision and pyloroplasty or by submucosal enucleation through a gastrostomy ([2]).
